# The Price of Progress: Modern Environmental Health Hazards in Africa

**DOI:** 10.1289/ehp.117-a257a

**Published:** 2009-06

**Authors:** Tanya Tillett

With the spread of industrialization to all areas of the globe, scientists and policymakers alike have voiced mounting concerns over the ability of developing countries to limit the public health impacts of unchecked development. African nations, for example, have experienced rapid urbanization as well as tremendous expansion in industry and technology in an attempt to raise living standards and keep pace with the global community. But this trend has also exposed these nations to numerous modern environmental health hazards (MEHHs)—health threats that tend to accompany rapid development in the absence of health and environmental safeguards. A review of the scientific literature suggests that such hazards have added considerably to Africa’s disease burden and are increasing in public health significance **[*EHP*** **117:863–870; Nweke and Sanders]**.

The authors wrote that ongoing exposures to emerging MEHHs may soon rival the contributions of more traditional hazards—including malaria, poor access to safe drinking water, and lack of basic sanitation—that have long troubled Africa. Furthermore, poor and malnourished populations may be more vulnerable to the impacts of MEHHs, given that malnutrition increases susceptibility to toxicologic challenges. According to the United Nations Industrial Development Organization, Africa’s pollution intensity (pollution generated per unit of production output) is among the highest in the world.

Reviewing published epidemiologic, exposure, and environmental studies of chemical agents, the researchers noted ongoing occupational and nonoccupational exposures to organochlorine pesticides such as DDT and to heavy metals such as lead and mercury. All these pollutants are known to persist in the environment and accumulate in the food chain over time, as confirmed by biomonitoring studies of farmworker populations in several African nations. The authors also presented evidence of existing and emerging air toxics issues related to both indoor and outdoor air pollution, pervasiveness of toxic chemicals in consumer products (such as arsenic and chromium in canned beverages), and inadequate management of domestic and industrial waste streams.

Relatively robust exposure data for lead showed elevated body burdens in African populations exposed to lead-bearing soil, dust, and paint, offering suggestive evidence of ongoing exposures to MEHHs at biological levels associated with adverse health impacts. Some studies found elevated body burdens of mercury in exposed populations such as miners, workers involved in ore processing, children who resided in mining communities, and women who habitually used soaps that contain high concentrations of inorganic mercury (such soaps are marketed as skin and hair lighteners). However, very few body burden studies have provided conclusive evidence of the relationship between heavy metal exposures and increased disease risks in African populations.

Management of MEHHs in many African nations has long been hampered by a lack of various safeguards for environmental health, such as stable institutions, adequate infrastructure, monitoring capacity, and regulatory frameworks. The researchers proposed that MEHHs should occupy a priority spot on Africa’s public health and policy agenda, and emphasized that future public health policy should consider these newer environmental health risks in tandem with other longstanding public health issues.

## Figures and Tables

**Figure:**
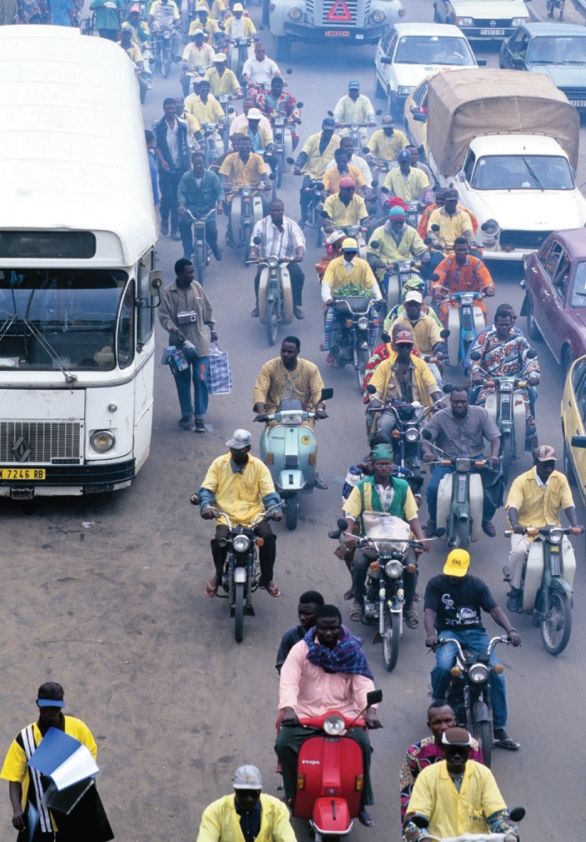
Leaded gas is now banned in sub-Saharan Africa but is still sold in some northern nations.

